# Reducing physical assaults on residents through implementation of project BETA: Best practices in the evaluation and treatment of agitation

**DOI:** 10.1002/aet2.11064

**Published:** 2025-02-14

**Authors:** Lynn Roppolo, Joshua L. Choe, Luke Beyer, Garrett Blumberg, David W. Morris, Jeffery Metzger, Jedidiah Leaf, Gilberto Salazar, Deborah Bishop‐Penn, A. J. Kirk, Christine Ramdin, Fuad Khan

**Affiliations:** ^1^ University of Texas Southwestern Medical Center Dallas Texas USA; ^2^ Parkland Health and Hospital System Dallas Texas USA; ^3^ John Peter Smith Health Network University North Texas & Texas Christian University Fort Worth Texas USA; ^4^ Rutgers University Newark New Jersey USA

**Keywords:** agitation, BETA, emergency, physical assault, residency, resident, workplace violence

## Abstract

**Background and objectives:**

We created a multitude of initiatives that were in line with the principles of the BETA (Best Practices in the Evaluation and Treatment of Agitation) guidelines to determine if these initiatives would reduce the physical assault rate by patients on emergency medicine (EM) residents.

**Methods:**

We conducted three cross‐sectional surveys of our EM residents (PGY‐1 to ‐3) to determine the incidence of physical assaults by agitated patients at a large county hospital emergency department. These were primarily anonymous REDCap surveys and were administered at the following intervals: (1) pre–BETA initiative implementation, (2) approximately 12 months after implementation, and (3) 5 years postimplementation. Unfortunately, the in‐person deescalation, self‐defense, and simulation training were canceled 2 years prior to the last survey due to COVID‐19. The second survey only looked at the incidence of physical assaults during the prior 6 months whereas the other two surveys evaluated the incidence of physical assaults since starting residency.

**Results:**

The survey response rates for the three REDCap surveys were 76% (50/66), 80% (53/66), and 71% (49/69), respectively. The percentage of EM residents who were physically assaulted per survey period were as follows: preimplementation cumulative assaults 28% (14/50), 12 months after implementation for 1 full academic year 11.3% (6/53), and postimplementation cumulative assaults during residency 5 years later 30.6% (15/49). The two independent‐samples proportions tests comparing the number of physical assaults *before* and approximately 12 months *after* all of these initiatives were implemented was significant (*p* = 0.032).

**Conclusions:**

An education and training curriculum designed to improve EM residents’ ability to manage agitated patients may reduce the incidence of physical assaults on them by patients in their care. However, the decrease in physical assaults after these initiatives followed by the increase in physical assaults experienced after the COVID‐19 pandemic are most likely multifactorial.

## INTRODUCTION

Workplace violence is defined as any act or threat of physical violence, harassment, intimidation, or other threatening disruptive behavior that occurs in the work environment.^1^ The emergency department (ED) is well known for being an environment at high risk for workplace violence and continues to be on the rise.^2^, ^3^, ^4^, ^5^, ^6^, ^7^ Several factors may contribute to this increase in workplace violence in the ED: overcrowding, inpatient boarding, long wait times, high stress levels, and staffing issues.^8^, ^9^, ^10^, ^11^ Patient volumes continue to rise as EDs account for almost half of hospital‐associated medical care delivered in the United States.^10^ The Emergency Medical Treatment and Labor Act (EMTALA) requires all EDs to provide a medical screening examination and stabilization to any individual who presents with a perceived emergency. Actual or potentially violent patients are brought to the ED if a medical evaluation is needed and EMTALA requires emergency physicians to care for them.^11^ Due to a national shortage of mental health resources, ED boarding of patients with acute mental health issues is also increasing and is another contributing factor to violence in the ED.^12^

Workplace violence is pervasive in all ED environments and employee assault is universal among all hospital sizes and in all types of communities.^13^ One of the many challenges faced by ED health care workers is the large spectrum of violent behaviors encountered in some ED patients such as use of profanity, threats, yelling, kicking, punching, and biting.^2^, ^3^, ^4^, ^5^ These violent encounters can occur during the patient's initial phases of care in the ED, a time at which limited information may be available to help determine the etiology of the agitation or violent behavior. In other instances, patients may be in a calm state or are less agitated and then abruptly escalate. Patients who exhibit violent behaviors almost always initially demonstrate varying levels of agitation which can be defined as excessive motor activity, verbal aggression, or physical aggression.^14^ Agitation often stems from a multitude of etiologies such as substance use or withdrawal, psychiatric or developmental conditions, or serious illness with or without delirium or psychosis.^4^, ^8^, ^15^ Severely agitated patients are at the greatest risk for violent behaviors and require an immediate assessment and management to mitigate the risk of injury to themselves or others. One common approach to managing a severely agitated patient in the ED has been to forcefully medicate and physically restrain them; however, this approach can have life‐threatening consequences not just from the potential for physical injury but from a cascade of physiological events in the patient that may ultimately lead to a severe metabolic acidosis and death.^16^

Limited comprehensive standardized treatment recommendations for managing agitation in the ED were available until consensus guidelines were published by the American Association of Emergency Psychiatry (AAEP). In 2012, the AAEP published the Best practices in Evaluation and Treatment of Agitation series in the *Western Journal of Emergency Medicine*, known as Project BETA, which addressed the critical need for an expert‐driven unified approach to managing agitated patients based on clinical experience and empirical research findings.^17^, ^18^, ^19^, ^20^, ^21^ Previous guidelines were primarily limited to medication strategies.^17^ The BETA guidelines focus on critical steps involved in the care of the agitated patient.^17^, ^18^, ^19^, ^20^, ^21^ Unfortunately, physical assaults on ED staff by patients in their care continue to contribute to workplace violence as guidelines such as BETA are not being uniformly implemented in the ED. In addition, there has been a paucity of literature that demonstrates the effectiveness of such guidelines.

We hypothesized that implementing the BETA guidelines into emergency medicine (EM) residency training and ED practice protocols would reduce physical assaults on our EM residents by improving their ability to proactively and safely manage agitated patients. Although physical assaults and workplace violence affect all ED staff, we focused only on our EM residents as this was a residency leadership education and training initiative and our finite resources limited our ability to study all staff in the ED.

## METHODS

We conducted three cross‐sectional surveys of a large 3‐year EM residency program (22 to 26 residents per class) to determine the incidence of physical assaults by agitated patients and related issues at our Level I trauma center and county hospital with over 220,000 ED patients visits a year. All EM residents at our program were recruited to participate. Participation was voluntary. All communications to the EM residents related to the study were primarily via email, group text, and in‐person verbal communications in the ED or at the weekly EM residency conference. Study data were primarily collected and managed using REDCap electronic data capture tools hosted at our university.^22^, ^23^ The residents were sent anonymous REDCap surveys at the following intervals: (1) pre–BETA initiative implementation, (2) 12 months after implementation of BETA initiatives, and (3) approximately 5 years postimplementation of BETA initiatives. The first and third REDCap surveys were obtained in October 2017 and May 2023, respectively. These two surveys contained very similar questions with only minor modifications in wording. The recall period for the physical assaults in the first and third surveys was since the beginning of their residency training and focused only on those physical assaults that took place in our county hospital ED (please refer to this link for the third survey).

The second survey obtained in December 2018 only included the following questions: postgraduate or PG year, if they had been physically assaulted since July 1 of that academic year, description of what happened, and thoughts on how it could have been avoided. The reason for obtaining physical assault data after July 2018 is that we wanted to ensure that all three EM residency classes had exposure to the BETA education and training initiatives *and* did not include the physical assaults that occurred *before* the training. Physical assaults on any EM resident were then closely tracked monthly through a variety of methods for the remainder of the 2018–2019 academic year. These methods included reporting by the residency and ED leadership, frequent group chats by one of the EM residents on our study team, and direct verbal communications. A REDCap survey was not repeated at the end of the 2018–2019 academic year as no physical assaults on any of the EM residents were identified through these other methods. A long‐term anonymous REDCap survey was also planned for June of 2021 to ensure that the graduating class experienced the BETA education and training initiatives for their entire residency but did not happen due to the COVID‐19 pandemic when all nonessential in‐person education and training was canceled. We conducted our final survey in May 2023 to ensure that all three of the residency classes received the 30‐min zoom lecture on the managing the agitated patient based on the BETA guidelines, which is currently given every 18 months and is the only training the residents receive on this topic at their weekly residency conference. Multiple reminders were sent to the residents over a 2‐week period to complete the surveys via group chats and email, during the weekly EM residency conference, and through personal communications. This study was approved by the university's institutional review board.

### BETA guideline initiatives to improve the management of agitated patients in the ED

#### Phase 1: Creating the ED agitation protocol and order set

A multidisciplinary committee was created at our county hospital with representation from the following: EM attendings and residents, psychiatry attendings who staffed the psychiatric ED, EM residency leadership, nursing leadership, pharmacy, toxicology, and police. We used the behavioral activity rating scale or BARS (Table 1) to stratify patients into different levels of agitation, although some preferred mild, moderate, and severe stratification of agitation severity.^24^

**TABLE 1:** BARS.^24^

BARS level	Description
7	Difficult to arouse
6	Asleep but can be aroused normally to verbal and physical contact
5	Drowsy, appears sedated
4	Normal level of activity
3	Increased overt physical and verbal activity; calms down with instruction
2	Continuously active and more difficult to calm down but not violent
1	Violent and needs restraint, unable to calm down

Abbreviation: BARS, Behavioral Activity Rating Scale.

**FIGURE 1:**
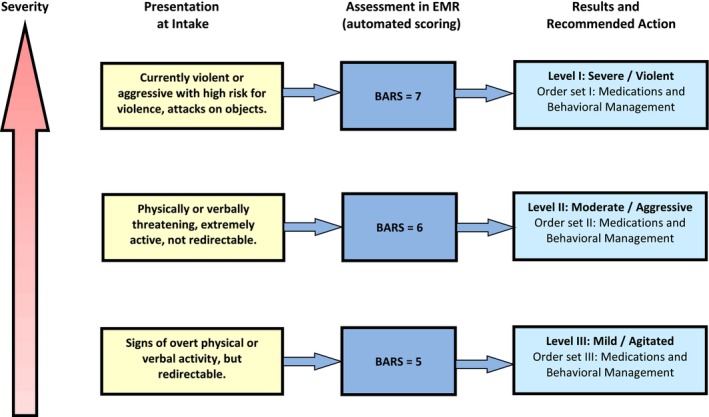
Violence/agitation severity leveling. BARS, Behavioral Activity Rating Scale; EMR, Electronic Medical Record.

This committee created a protocol for managing agitated patients presenting to the ED and an ED agitation order set in the electronic health record (EHR). The general framework of the agitation protocol and order set in the EHR is outlined in Figure 1 and Table 2, respectively.^25^ The recommended protocol for approaching the “severely” agitated patient was as follows:

1. Patient is triaged directly to the critical care area of the ED and evaluated by an emergency physician.
2. Law enforcement is immediately called to the patient's bedside to ensure safety of the patient and all ED staff. All patients are screened for weapons with portable metal detectors.
3. Verbal deescalation is attempted first, if possible, by police, the emergency physician, or other ED staff.
4. If deescalation attempts are not effective and the patient is severely agitated with potential for injury to self or others, forced medication and physical restraint are initiated as follows.
  Five law enforcement officers physically restrain the patient with one at the head of the bed and the others at each extremity and remain at the bedside until the patient is no longer a threat to the ED staff.
  - The nurse prepares the medication ordered by the physician for intramuscular (IM) administration and waits at the bedside until law enforcement informs them that it is safe to give the IM injection.
  - The physician orders additional medications, diagnostic studies, physical restraints, etc., as indicated. The physician continuously assesses the patient from a distance and ensures the patient's safety (e.g., physical restraint is not causing injury or interfering with the patient's ability to breath). The physician and other staff distance themselves from the patient at all times until it is considered to be safe.
  - Once the patient is less agitated, the patient is positioned for safety (preferably on their back in a semirecumbent position). Wrist and ankle restraints are applied if needed, and monitors are placed (e.g., oxygenation, cardiac, capnometry). An IV is inserted and diagnostic studies are obtained as indicated. A sitter is at the bedside at a safe distance for the entire duration that the patient is in the ED and the patient is closely monitored by nursing staff. Sitters carry radios to quickly call for assistance if needed.

**TABLE 2:** Outline of agitation order set in electronic medical record.

	Orders	Description or comment
1	Agitation assessment levels with reminders of how to deescalate	Levels of agitation described corresponding to “mild” or BARS 5, “moderate” or BARS 6, and “severe” or BARS 7.
2	Direct observation	One to one for those at risk for violent behavior such as those with severe agitation; police were at the bedside for incarcerated patients or other higher risk patients.
3	Physical restraints	Nonviolent or soft restraints versus violent or leather restraints.
4	Medications	Two main categories: psychosis present or undifferentiated agitation. Under each main category, medications were specified by the severity of the agitation with recommended doses, routes, and warnings such as a dose reduction for the elderly.
5	Nursing	IV access, monitoring, when to notify the physician.
6	IV fluids	IV orders with and without additives.
7	Diagnostic studies	To evaluate for causes of agitation (e.g., traumatic head injury, thyroid studies, basic labs, toxicology screen, creatine phosphokinase, or CPK).

Abbreviation: BARS, Behavioral Activity Rating Scale.

#### Phase 2: Training EM residents in agitation management

The EM residents were given an annual 30‐min didactic lecture at their weekly EM residency conference on the “emergency management of agitation,” which focused on the BETA guidelines and included a review of the ED agitation protocol and the ED agitation order set. Each year, one of the monthly simulation labs included a 30‐min station with a simulated scenario focused on how to manage a severely agitated patient, which included verbal deescalation techniques, safe physical restraint, and pharmacologic interventions. Additionally, starting the following academic year, all EM interns received a modified (2‐hour) version of the deescalation and self‐defense training that the hospital offered to other ED staff (e.g., nurses). It was emphasized that agitation should be considered a high‐risk presentation, deescalation should always be the first intervention considered, calling for help early (e.g., law enforcement assistance), and physical restraint and involuntary medication administration should be a last resort.

Of note, several new policies and protocols were developed concurrently around the same time period by the hospital administration to improve safety and the care provided to agitated patients. These and some preexisting related practices are summarized below that further support the BETA initiatives.

1. Hospital's “zero tolerance” for violence policy is posted at hospital entrances.

2. All ED nurses receive training in deescalation and self‐defense training called SAMA (Satori Alternatives to Managing Aggression).^26^

3. The hospital's own law enforcement officers are stationed at all public entrances to the ED and screen all patients and visitors with metal detectors. One of the officers on duty is designated to be the LIFE (Law Enforcement Intervention for Environmental/Patient Safety) officer who has advanced training in communication and deescalation skills.^27^ This specially trained officer may familiarize themselves with potentially violent patients present in the ED and can easily return if a patient starts to escalate. Hospital law enforcement aids in physical restraint when indicated. Cameras are located throughout the ED and law enforcement used body cameras to record events.

4. ED staff can contact police by radio or phones or use of a panic button located in each patient care area.

5. Nurses in triage screen patients with the violence screening tool STAMP (Staring and eye contact, Tone and volume of voice, Anxiety, Mumbling, and Pacing) and document this in the EHR where it is easily visible on the patient tracking board.^28^ Prior history of violent behavior is also highlighted in the EHR. A dedicated wrist band is placed on the patient and a sign is placed on their ED door entrance to their room to alert health care workers of the violence risk.

6. The clothing of all patients who are formally detained on an emergency mental health detention (for acutely potentially dangerous behavior or ideations to themselves or others) are removed and these patients are dressed in green hospital gowns with yellow socks for easy identification. Their belongings are searched and secured in the presence of law enforcement who also assist with changing the patient's clothes to ensure safety. All objects are removed from the patient's room if possible and placed in a secured storage container.

7. There is an ED Violence Prevention Response Team (VPRT), composed of ED leadership, nurses, and law enforcement officers, who round on potentially violent patients and may be called to the bedside during any escalations. After an event, the VPRT does a debriefing to prevent recurrences. The VPRT mostly respond to significant events but are not a team who gets “activated” or called upon to respond to every escalating or severely agitated patient like behavioral response teams discussed below.

For clarification, we used the BARS to differentiate between patients who were mildly, moderately, or severely agitated during the lecture and simulation training and in the agitation order set but this was not uniformly used by the nurses and physicians who sometimes preferred to use “mild, moderate, or severe”. STAMP was implemented by the hospital and only performed at triage by nursing staff. If a patient was STAMP positive, this was noted on the track board next to the patient's name to indicate that the patient was at risk for violent behavior. STAMP was not an assessment that differentiated between mild, moderate or severe agitation. Patients were STAMP positive or not STAMP positive. Due to COVID‐19 gathering restrictions, all in‐person training was canceled in March 2020 and gradually resumed once the pandemic was over. The only formal BETA guideline–based education that the residents received after COVID was a 30‐min zoom lecture every 18 months.

### Statistical analysis

Descriptive statistics were used to summarize all data. The chi‐square difference of proportions test or Fisher's exact test were used as applicable to compare proportions. All significant *p*‐values are reported with an asterisk. All *p*‐values were reported at the 0.05 significance level.

## RESULTS

The survey response rates for the three anonymous REDCap surveys were pre–BETA implementation 76% (50/66), 12 months post–BETA implementation 80% (53/66), and 5 years postimplementation 71% (49/69; see Table 3 for survey results for the pre‐ and 5 years post–BETA initiative implementation). Two additional questions asked under “patient factors” in the first survey, which accounted for 18% under “other” patient factors, were as follows: inability to deal with crisis situations (14/50, 28%) and gang involvement (4/50, 8%). These were eliminated from the 5‐year survey in an effort to shorten the survey and were thought to not contribute a significant amount to our understanding of patient factors. The choice for “other” patient factors was also offered in the first survey but none of the 50 residents checked that response. Only one resident checked the “other” response for patient factors in the 5‐year survey.

**TABLE 3:** Comparison of survey data before and five years after BETA initiatives implementation.

Survey question	Answers choices for each survey question	Pre‐BETA initiative survey (*n* = 50)	5 years post‐BETA initiative survey (*n* = 49)	*p*‐Value
Gender	Male	35 (70.0%)	28 (57.1%)	0.09
	Female	15 (30.0%)	21 (42.9%)	0.09
Resident level	R1	17 (34.0%)	18 (36.7%)	0.39
	R2	20 (40.0%)	17 (34.7%)	0.29
	R3	13 (26.0%)	14 (28.6%)	0.39
Years experience caring for significantly agitated patients	Less than a year	13 (26.0%)	10 (20.4%)	0.25
	1‐2 years	22 (44.0%)	22 (44.9%)	0.46
	3‐5 years	10 (20.0%)	16 (32.7%)	0.08
	More than 5 years	4 (8.0%)	1 (2.0%)	0.36
	No prior experience	1 (2.0%)	0	1
How many times have you been physically assaulted in the ED as an EM resident?	Never	36 (72.0%)	34 (69.0%)	0.39
	Once	9 (18.0%)	7 (14.3%)	0.31
	Twice	3 (6.0%)	5 (10.2%)	0.49
	Three times	2 (4.0%)	2 (4.1%)	1
	Four times	0	1 (2.0%)	0.49
How many times have you seen someone else physically assaulted in the ED as an EM resident?	Never	16 (32.0%)	14 (28.6%)	0.36
	Once	8 (16.0%)	6 (12.2%)	0.30
	Twice	11 (22.0%)	10 (20.4%)	0.42
	Three times	5 (10.0%)	7 (14.3%)	0.26
	Four times	4 (8.0%)	6 (12.2%)	0.52
	Five times	2 (4.0%	2 (4.1%)	1
	Seven or more	4 (8.0%)	4 (8.2%)	1
PATIENT factors that may have contributed to the physical assault (s)	Alcohol	29 (58.0%)	19 (38.8%)	0.03*
	Drugs	40 (80.0%)	31 (63.3%)	0.03*
	Psychiatric disease	37 (74.0%)	35 (71.4%)	0.39
	Organic brain syndrome or dementia	13 (26.0%)	19 (38.8%)	0.09
	Other	18 (36.0%)	1 (2.0%)	0.0001*
STAFFING factors that may have contributed to the physical assault (s)	Lack of adequate staff	14 (28.0%)	12 (24.5%)	0.36
	Delay in patient assessment	Not asked	3 (6.1%)	–
	Delay in patient management (de‐escalation, calming medications)	Not asked	19 (38.8%)	–
	Lack of information about patient (e.g. violence history or risk factors for violent behavior)	16 (32.0%)	17 (34.7%)	0.39
	Lack of violence prevention training	8 (16.0%)	9 (18.4%)	0.38
	Actions by staff that were thought to increase patient's agitation (e.g. refusing to address patient requests, being confrontational)	Not asked	14 (28.5%)	–
	Other	6 (12.0%)	1 (2.0%)	0.11
ENVIRONMENTAL or hospital‐based factors that may have contributed to the physical assault (s)	Overcrowding in the ED or long wait times	23 (46.0%)	25 (51.0%)	0.31
	Lack of security/police support or did not respond in a timely manner	11 (22.0%)	10 (20.4%)	0.42
	Lack of metal detectors/alarms	2 (4.0%)	9 (18.4%)	0.03*
	Lack of policies/procedures for handling “known” violent offenders	12 (24.0%)	20 (40.8%)	0.04*
	Lack of a protocol to “assess” for agitated and potentially violent patients in the ED	Not asked	20 (40.8%)	–
	Lack of a protocol to “manage” for agitated and potentially violent patients in the ED	Not asked	19 (38.8%)	–
	Other	3 (6.0%)	1 (2.0%)	0.62
How often do you feel safe while working in the ED	Often or always	45 (90.0%)	41 (83.7%)	0.39
How satisfied are you with security/police?	Somewhat satisfied	16 (32.0%)	22 (44.9%)	0.09
	Very satisfied	24 (48.0%)	24 (49.0%)	0.46
What would make you feel safer while working in the ED?	Behavioral response team	28 (56.0%)	30 (61.2%)	0.30
Comfort level with managing severely agitated patients?	Very uncomfortable	4 (8.0%)	0	0.12
	Somewhat uncomfortable	7 (14.0%)	5 (10.2%)	0.76
	Neutral	8 (16.0)%	9 (18.4%)	0.80
	Somewhat comfortable	23 (46.0%)	30 (61.2%)	0.06
	Very comfortable	8 (16.0%)	5 (10.2%)	0.55

*Note*: Both deescalation/self‐defense training and residency conference simulation session canceled 2 years prior to 5‐year survey. All significant *p*‐values are reported with an asterisk (*). All *p*‐values were reported at the 0.05 significance level.

The survey obtained 12 months after all BETA initiatives implemented (in December 2018) focused only on physical assaults by patients on EM residents since July 2018 and revealed an 11.3% (6/53) physical assault rate. The details of these physical assaults described in the survey are summarized in Table 4. None of the interns were physically assaulted in this survey and none of the residents were assaulted more than once. The respondents included 34.0% (18/53) PGY‐1s, 34.0% (18/53) PGY‐2s, and 32.1% (17/53) PGY‐3s. There were no reports of any EM resident physical assaults through our monthly surveillance for the remainder of the 2018–2019 academic year.

**TABLE 4:** Description of physical assaults from December 2018 survey data.

EM resident year in training	Description of physical assault by EM resident
PGY‐2	*Was slapped in the face by an old lady who I think was sundowning while pumping up her bed*.
PGY‐3	*Patient walked behind the desk and got increasing closer. Things thrown at me. Felt threatened*.
PGY‐3	*I was 20 minutes into my 12‐hour shift which began at 7 AM. Four police were walking a patient down the hallway in a green gown. He was not showing any signs of aggression. He suddenly yelled out “I want another evaluation!” He ran around the nursing counter and as I stood up to confront him, he wrapped his arm around my neck and put me in a choke hold. It took around 30–40 seconds for the police to get him off of me*
PGY‐3	*Was asking patient if she could move her leg. Patient said yes and kicked her leg up in the air from the bed and hit me in the temple. Patient was not altered, and later apologetic saying she did it as a joke*.
PGY‐3	*Patient known psych patient. Jumped out of bed and swatted at me*.
PGY‐2	*Patient did not want to return to jail after negative workup. Staff and officers present attempted to physically restrain. I was kicked in shoulder. No meds given. Patient removed with officers*.

Abbreviation: PGY, postgraduate year (in training).

As noted in Table 3, the percentage of EM residents who were physically assaulted in the pre‐ and 5 years post–BETA initiative implementation surveys were 28.0% (14/50) and 30.6% (15/49), respectively, which was not significantly different. The two independent samples proportions test comparing the number of physical assaults *before* (28.0%) and approximately 12 months *after* (11.3%) these initiatives were implemented was significant (*p* = 0.032). Of note, several of the residents reported that they were physically assaulted several times in the pre‐ and 5 years post–BETA guideline initiatives implementation. If physical assault rates were calculated by the actual number of physical assaults on an EM resident instead of what percentage of EM residents were physically assaulted, the pre‐ and 5 years post–BETA guideline initiative implementation physical assault rates would have been 42.0% (21/50) and 55.1% (27/49), respectively. None of the residents were physically assaulted multiple times in the 12‐month period after the BETA guideline initiatives implementation (2018–2019). The three most common underlying patient conditions related to the physical assault were alcohol use, drug abuse, or a psychiatric disorder. Most of the residents felt safe and were satisfied with law enforcement in the ED, but their comfort level with taking care of severely agitated patients was highly variable. None of the physical assaults on the EM residents during the study period caused any significant physical injury.

## DISCUSSION

Project BETA provides practical guidelines based on available evidence and a consensus panel of experts on agitation management. These practices are easy to teach and use at the bedside. Most importantly, they are effective in reducing physical assaults by agitated patients on ED staff as demonstrated by the reduction in physical assaults on our EM residents 12 months after implementation of several BETA guideline initiatives. The annual physical assault rate of 11.3% on our EM residents by patients in the ED is the lowest annual physical assault rate ever reported in the literature. This is much lower than the 30.8% physical assault rate over a 6‐month period that was recently reported by McGuire et al.^3^ Our study took place at the busiest single facility ED with the largest single‐class EM residency program in the United States. Unfortunately, this reduction in physical assaults was not sustainable and eventually increased to a level that approximated our pre–BETA guideline initiative implementation levels.

The two in‐person EM residency BETA guideline training initiatives were not resumed after COVID as the faculty who initiated and coordinated this training left the residency leadership and no one assumed this role. The increase in physical assaults demonstrated in the 5‐year postinitiative survey was slightly higher than our preimplementation physical assault rates and may be attributed to the discontinuation of this important in‐person training, which reinforces important BETA principles such as deescalation.^29^ The 5‐year survey also demonstrated that many of the residents were not aware of the ED agitation protocol or order set. This may be partly related to their limited training in agitation management as their scheduled education on this topic after COVID‐19 was limited to a 30‐min lecture every 18 months at the weekly residency conference. The cognitive overload that can happen from all of the EM core content received on conference days and absence at that particular conference due to other clinical rotations, illness, and vacations are some plausible additional factors affecting how much of the information conveyed in this lecture format is actually retained. Furthermore, some may not have known or may have forgotten that the ED agitation order set and protocol even existed as this was not a hospitalwide project that required periodic retraining like other annual compliance content. Although the discontinuation of in‐person training for our residents after COVID may have increased their risk for being physically assaulted by patients, the cause is most likely multifactorial. Nursing shortages, inexperienced staff, ED crowding, and an increase in the number of patients at risk for agitation are some examples of possible unmeasured contributors.

Agitation is not a diagnosis but a behavioral symptom caused by a multitude of etiologies that can progress to violence. Despite efforts made by national organizations and the wide distribution of published guidelines to improve agitation management in the ED, the reported statistics of verbal and physical assaults to ED staff continue to be alarming.^3^, ^4^, ^5^, ^8^, ^30^ The accuracy of these data may be limited due to recall bias, underreporting, or even increased reporting, which may have inflated these numbers, but the shocking reality is that physical assaults by patients on ED staff continues to exist. Our survey responses from EM residents suggests that a delay in patient management and lack of information related to violence risk factors were the two most common staffing factors contributing to physical assault. Patients presenting with agitation to the ED need to be prioritized in the same manner as other life‐threatening presentations. Delays in evaluating these patients, failure to recognize their violence risk, issues with concurrent medical conditions, and complications related to agitation management can have devastating immediate and long‐term consequences.^15^, ^19^, ^20^, ^31^ Severely agitated patients need to be promptly placed in an appropriate location in the ED with enough resources to reduce the incidence of self‐harm and injury to others. Law enforcement or other trained individuals should be present in the ED and be immediately at the bedside of any patient who poses a violence risk. Our residents reported in the survey that they felt safe and supported by our law enforcement in managing agitated patients but this valuable resource needs to be called upon early to mitigate escalation of a patient's agitation and to protect our health care providers from harm. Physical restraint should not be the responsibility of ED staff as doing so places them at high risk for serious physical injury.

Physical assault is the most concerning and dangerous form of hospital‐based workplace violence, which may be prevented by uniformly implementing evidence‐based guidelines like BETA.^29^ Verbal deescalation is a powerful tool that can be taught and performed by any member of the health care team. Unless the patient is actively violent, verbal deescalation should be attempted first.^18^ This simple intervention may significantly reduce a patient's agitation and help them regain control of their behavior and even agree to take oral medication to reduce their agitation symptoms if needed.^32^

The critical decision to physically restrain and administer involuntary medication should be of last resort.^33^ This depends on the severity of the patient's agitation after deescalation is attempted, the underlying etiology of the patient's agitation, and their clinical presentation. Our resident survey revealed that the vast majority of patients who physically assault them were intoxicated with alcohol or drugs, had a psychiatric disorder, or have a history of organic brain syndrome/dementia. This suggests that these patients may be difficult to verbally deescalate and that physicians should have a lower threshold to provide anxiolysis or sedation to prevent escalation to a violent and combative state. Pharmacologic intervention should always be administered to “calm” the severely agitated patients who require physical restraint and should not be referred to as a “chemical restraint.”^18^ Caution should always be taken to minimize any adverse effects from the medications like oversedation and any injury to the patient or ED staff during administration. Physical restraint can not only cause physical injury to the patient and ED staff but studies have demonstrated negative psychological effects on patients such as posttraumatic stress syndrome.^34^, ^35^

Our final survey demonstrated that the most common intervention that our EM residents thought would make the ED safer was having a behavioral response team. Behavioral Emergency Response Teams (BERTs) are composed of a group of specially trained individuals who rapidly respond to patients whose agitated behavior is escalating and can reduce physical restraint use.^36^ Wong et al.^37^ reported on the effectiveness of a structured agitation response team, which reduced physical restraint use over a 5‐year period. A recent pilot study using simulation by Duncan et al.^38^ found a reduced physical restraint use and improved confidence in managing agitation by ED staff. In addition, creating protocols and order sets on the EHR can be helpful in implementing the current recommendations in agitation management by the entire health care team.^39^ Including guidelines for medications to reduce agitation may promote safe, efficient, and evidence‐based patient care.^40^ There are several newer medication recommendations for severely agitated patients that replace older pharmacologic models such as the “B52” cocktail (i.e., haloperidol, lorazepam, and diphenhydramine) that was frequently used and is no longer recommended. There is no single medication class that is most appropriate for all cases of agitation.^21^ Regardless of the class, the preferred route for the medication should be orally, ideally in a cooperative patient with the goal of maintaining a calm state in the ED without inducing sleep.^21^ IM injections are reserved for severely agitated patients and should be administered only when safe to do so.

Once a patient's agitation symptoms are controlled, they need to be medically cleared and dispositioned from the ED. One study found that over half require admission but many can be medically cleared and discharged.^41^ Project BETA is a practical solution that can be added to our armamentarium to not only improve the quality of care we deliver to these patients but also to potentially reduce the stigma of workplace violence in our ED environment and decrease the incidence of physical assaults on our own.

## LIMITATIONS

There are several limitations to this study. This was a single‐site, voluntary survey study that has many limitations such as selection bias, recall bias, and underreporting. There were multiple residents from different classes for a study over a 5‐year period, in a 3‐year residency program. The residents were a heterogenous and changing study group as the population surveyed in each time period was a different group of residents with different backgrounds and clinical experiences. There was also some variability in how residents were trained as only the new interns received the SAMA deescalation and self‐defense training and it was assumed that the upper levels had this training during the previous year. We did not collect data on which residents attended the residency conference lectures and simulation training as residents are sometimes excused for clinical duties and vacation. We also did not conduct a pre‐ or posttest after these trainings to determine their effectiveness. The physical assault rate on our residents was closely monitored during the year following implementation of the BETA guideline initiatives so the Hawthorne effect may have influenced our results.^42^ It was not known how often the agitation order set was used but it was clear that many residents were not aware it even existed in the 5‐year survey. The change in the incidence of physical assaults was most likely multifactorial. Several hospital initiatives to reduce workplace violence were taking place at the same time. There were also several other factors that were not accounted for that could also influence the increased physical assault rate amongst our EM residents such as the COVID‐19 pandemic, the number of agitated patients presenting to the ED, and the variability in individual practices of supervising attendings and support staff. One recent study found that ED volumes fell during the pandemic but ED crowding increased after the pandemic especially in psychiatric patients, which is similar to what happened at our county hospital and may contribute to an increase in physical assaults by patients on our EM residents.^43^ The second REDCap survey only focused on physical assaults from the first 6 months of the academic year after all BETA guideline initiatives were fully executed, with very close surveillance by several means at frequent intervals for the remainder of the academic year. Although unlikely, it was still possible that not doing a REDCap survey at the end of the 2018–2019 academic year could have missed physical assault incidents on our residents. We also did not stratify the assaults by year in residency, but the results would have most likely mirrored the findings published by Querin et al.^5^ The low physical assault rate among the intern class may have been due to interns spending less time spent in the ED and because they most likely would not have been tasked with caring for severely agitated patients. Finally, the lead author and principal investigator of this study was instrumental in creating these BETA guideline initiatives. She was part of the residency leadership until just before the COVID‐19 pandemic, which may have influenced the awareness of and compliance with these recommendations.

## CONCLUSIONS

An education and training curriculum designed to improve emergency medicine residents’ ability to manage agitated patients may reduce the incidence of physical assaults on them by patients in their care. However, the decrease in physical assaults after these initiatives followed by the increase in physical assaults experienced after the COVID‐19 pandemic are most likely multifactorial.

## AUTHOR CONTRIBUTIONS

Study concept and design (Lynn Roppolo, Luke Beyer, Garrett Blumberg, David W. Morris, Fuad Khan, Jeffery Metzger, Deborah Bishop‐Penn, AJ Kirk). Acquisition of the data (Lynn Roppolo, Luke Beyer, Garrett Blumberg, Jedidiah Leaf). Analysis and interpretation of the data (Lynn Roppolo, Christine Ramdin). Drafting of manuscript (Lynn Roppolo, Joshua L. Choe, David W. Morris). Critical revision of manuscript (Lynn Roppolo, Joshua L. Choe, LBL, David W. Morris, Jeffery Metzger, Jedidiah Leaf, Gilberto Salazar, Deborah Bishop‐Penn, AJ Kirk, Fuad Khan), Statistical Expertise (Christine Ramdin), and Acquisition of Funding (none).

## CONFLICT OF INTEREST STATEMENT

The authors declare no conflicts of interest.
